# DIURNAL CORTISOL SECRETION AND SELF-REPORTED AND CAREGIVER-REPORTED QUALITY OF LIFE IN PEOPLE LIVING WITH DEMENTIA

**DOI:** 10.1093/geroni/igac059.2313

**Published:** 2022-12-20

**Authors:** Fanghong Dong, Subhash Aryal, Nancy Hodgson

**Affiliations:** University of Pennsylvania, Philadelphia, Pennsylvania, United States; University of Pennsylvania, Philadelphia, Pennsylvania, United States; University of Pennsylvania, Philadelphia, Pennsylvania, United States

## Abstract

**Introduction:**

People living with dementia (PLwD) report lower quality of life (QoL), compared to healthy older adults. The poorer QoL is not fully accounted for by the severity of dementia. Dementia is associated with prominent neuroendocrine changes, however, there is a lack of research examining whether biological factors are related to QoL in PLwD. This study examined relationships between cortisol, symptom severity, and QoL in PLwD.

**Methods:**

A total of 143 participants aged 55-94 years (65.7% women) in the Healthy Patterns Study (NCT03682185) provided three saliva samples at wake-up (AM1), 30 minutes (AM2) after waking, and bedtime (PM) on two consecutive days. We derived cortisol awakening response (CAR), wake to bedtime cortisol slope, and diurnal mean cortisol secretion. Sociodemographic and severity of dementia were assessed by interviews and questionnaires. Self-reported and caregiver-reported QoL was measured using the Quality of Life in Alzheimer’s Disease (QoL-AD).

**Results:**

Poorer QoL was associated with more severe dementia rating. Flattened cortisol slope was significantly correlated with overall poorer self-reported QoL (β=0.43, p=0.017), but not caregiver-reported QoL (p=0.12), after controlling for severity of dementia and demographic variables. We did not find a significant relationship between CAR and diurnal mean cortisol with QoL.

**Conclusions:**

This study provides novel evidence linking neuroendocrine mechanisms to QoL in PLwD. The findings indicate that dysregulation of the hypothalamic-pituitary-adrenal axis is linked to poorer QoL, independently of the severity of dementia. Biopsychosocial approaches to QoL for PLwD may lead to a greater understanding of the underlying mechanisms.

